# Anatomical Features and Morphometric Characteristics of the Sphenoidal Sinus in MRI Studies

**DOI:** 10.7759/cureus.66764

**Published:** 2024-08-13

**Authors:** Kristian Bechev, Nina I Yotova, Kostadin Kostadinov, Ilko Ilyov, Daniel Markov

**Affiliations:** 1 Neurological Surgery, Universitetska Mnogoprofilna Bolnitsa za Aktivno Lechenie (UMBAL) Pulmed, Plovdiv, BGR; 2 Anatomy, Medical University of Plovdiv, Plovdiv, BGR; 3 Environmental Health, Research Institute, Medical University of Plovdiv, Plovdiv, BGR; 4 Social Medicine and Public Health, Faculty of Public Health, Medical University of Plovdiv, Plovdiv, BGR; 5 Medicine, Medical University of Plovdiv, Plovdiv, BGR; 6 General and Clinical Pathology, Medical University of Plovdiv, Plovdiv, BGR

**Keywords:** pneumatization of the sphenoid sinus, normal morphology of the sphenoid sinus, transsphenoidal access to the skull base, magnetic resonance imaging of the brain, sphenoid sinus

## Abstract

Differential access to pathological sellar processes and adjacent regions is determined by the anatomic structures identified through diagnostic imaging. Both direct endonasal access (microscopic or endoscopic) and sublabial access utilize the sphenoid sinus (SS) as the primary surgical pathway. Critical factors include the pneumatization of the sinus, its intermediate septa, and the presence of a double wall, consisting of a connective tissue membrane along the dorsal wall of the SS.

The present study aims to demonstrate the significance of the size and type of the SS based on MRI measurements. The type of SS, its pneumatization, and the proximity of adjacent brain structures are crucial for different surgical approaches to the SS and pituitary fossa. In neurosurgical practice, six main types of sinuses are recognized: sphenoid body type, lateral type, clival type, lesser wing type, anterior type, and combined type. Failure to consider these variations can lead to damage to the cavernous sinus, Meckel's cave, nerve structures in the middle cranial fossa, planum sphenoidale, suprasellar region, and vital brainstem structures located on the clivus.

Randomly included MRI measurements were conducted on 112 patients from Pulmed University Hospital, Plovdiv, Bulgaria, categorized into two cohorts based on gender, with mean ages of 51 years for men and 47.8 years for women. The measurements, recorded in centimeters, were obtained using two imaging software programs, RadiAnt DICOM Viewer (Medixant, Poznan, Poland) and Weasis DICOM Viewer (Nicolas Roduit, https://github.com/nroduit/Weasis). No statistically significant differences were observed between the measurements produced by the two programs.

Measurements of the SS were taken in two equal groups, using three different projections: axial, sagittal, and coronal. The results for height, width, and depth showed average sizes of 2.73-3.04 cm in axial projections, 1.70-2.64 cm in sagittal projections, and 2.86-3.03 cm in coronal projections. The minor differences between axial and coronal measurements of the same parameters (height and width) are statistically acceptable and attributed to the varying angles of the MRI scans. These measurements are crucial for planning surgical access to the sellar and parasellar regions, determining the necessary bony resection of the posterior wall of the SS, and preventing complications from excessive bony trepanation.

## Introduction

This clinical study aims to elucidate the complex anatomy of the sphenoid sinus (SS), pituitary, and parapituitary regions and its implications for surgical planning using MRI of the brain. The endoscopic transsphenoidal approach to the base of the temporal lobe has become a prevalent technique in neurosurgical practice, necessitating detailed studies on the microanatomy of the region. Numerous studies have explored the SS volume, width, height, and depth. In this study, we demonstrated that the width of the SS also determines the larger intercarotid space and no statistical correlation was found between men and women. This fact is because when the SS is pneumatized more laterally, the internal carotid artery is displaced more laterally, and thus the distance between the two blood vessels increases. 

This study enabled us to corroborate existing literature and categorize the SS into six main types: sphenoid body type, lateral type, clival type, lesser wing type, anterior type, and combined type [1-3]. The MRI of the brain provides crucial information regarding the tumor's location relative to normal anatomical structures in this area. The width of the SS is particularly important for preoperative planning of the bony trepanation of the sinus, helping to avoid iatrogenic damage to the carotid artery. We demonstrated a correlation between the width of the SS and the intercarotid distance, which has an immediate relationship with the types of the SS and its width. The MRI imaging of the skull base has become a standard preoperative preparation tool for patients with tumors and inflammatory diseases affecting the SS and pituitary region.

## Materials and methods

This study was conducted at the Department of Neurosurgery, University Hospital Pulmed, Plovdiv, between January 3, 2023, and January 3, 2024. A total of 112 patients participated in the study, divided into two groups of men and women (the latter was done to see if there was a statistically significant difference between the two sexes), with a mean age between 47 and 50 years. The measurements were obtained using two imaging programs, RadiAnt DICOM Viewer (Medixant, Poznan, Poland) and Weasis DICOM Viewer (Nicolas Roduit, https://github.com/nroduit/Weasis). Patients were selected randomly from all MRI scans performed during the study period, with inclusion criteria ensuring the presence of coronal slices, Bulgarian ethnicity, normal morphology of structures in the area of interest, and inclusion of all types of SS.

The MRI scans of the brain were performed on all patients, and measurements were specifically conducted in the coronal plane of T2 projections to detail the anatomy of the SS and pituitary region. Measurements of the SS were conducted using two imaging programs, RadiAnt and Weasis, to minimize measurement errors.

Statistical analysis, including t-tests for comparing measurements between the two groups and correlation analysis to examine relationships between SS dimensions and intercarotid distance, was performed using R software (The R Core Team, R Foundation for Statistical Computing, Vienna, Austria). The Shapiro-Wilk test was used to assess the normality of the data before conducting further statistical analyses. Descriptive statistics such as mean and standard deviation were calculated to summarize the dimensions of the analyzed structures.

Ethical considerations were strictly adhered to in accordance with the ethical standards of the institutional and national research committee and the Declaration of Helsinki. Verbal informed consent was obtained from all participants prior to their inclusion in the study.

**Figure 1:**
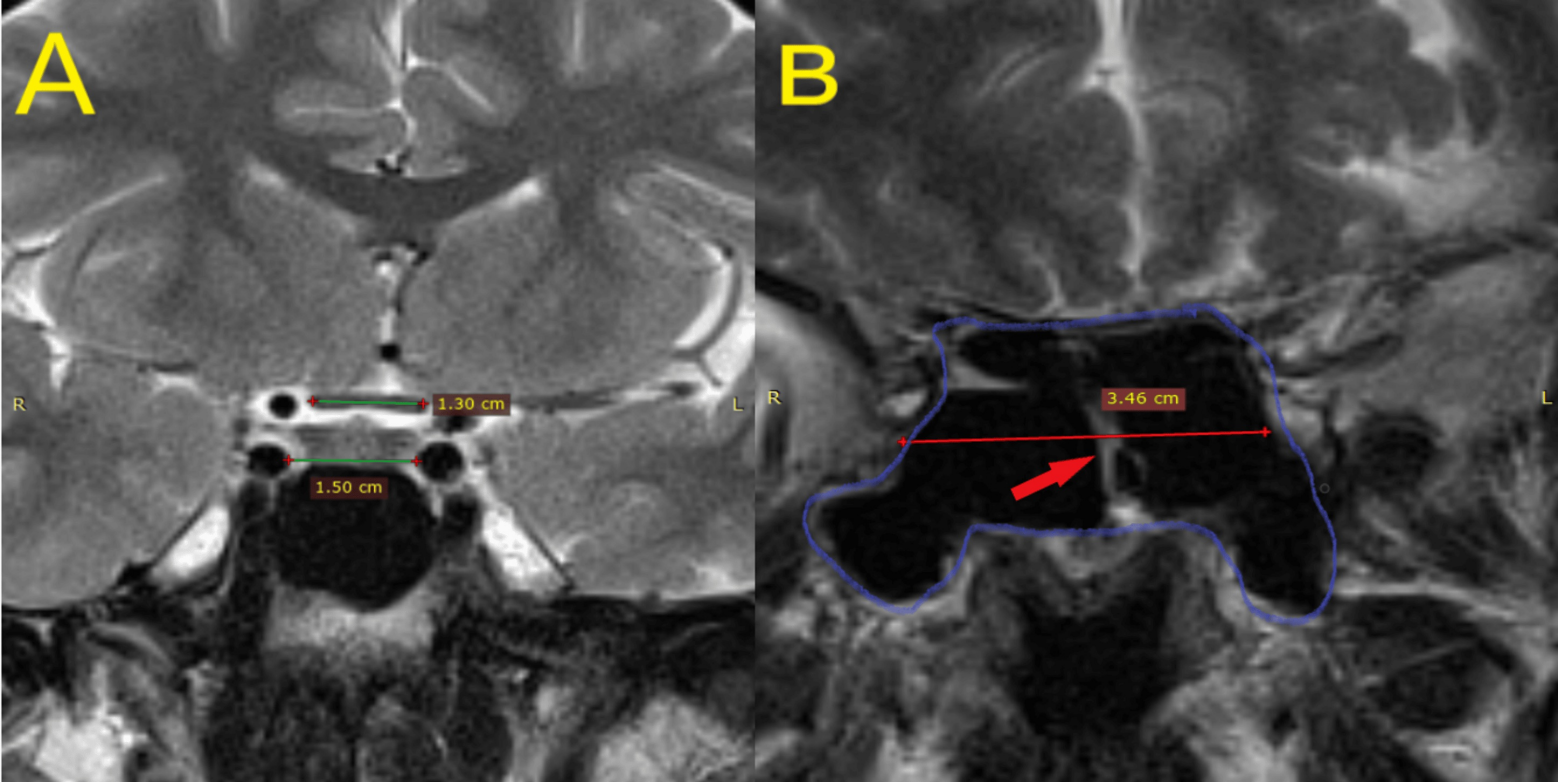
An MRI image of the brain (cortical section in T2 sequence) Panel A demonstrates the measurement of the width of the optic chiasm and intercarotid space. The measurement is taken horizontally across the widest point of each structure; Panel B demonstrates the measurement of the width of the sphenoid sinus (the part circled in blue represents the sphenoid sinus) and the medial septum (red arrow). The measurement of the sphenoid sinus width is taken horizontally across its widest point, while the medial septum's width is measured similarly.

Figure 1 illustrates coronal sections through the intercarotid space, chiasm, and SS, demonstrating the methodology used for the measurements and providing visual context to the study's findings.

## Results

The results are summarized in Table 1, detailing the descriptive statistics and 95% confidence intervals for key variables.

**Table 1:** Descriptive statistics and 95% confidence intervals for study variables Note: The CI of the mean assumes sample means follow a t-distribution with N -1 degrees of freedom

Variable	Mean	95% confidence	Interval	Median	SD	Shapiro-Wilk
Age	49.1	45.8	52.4	48.5	17.9	-
Male (%)	40.2	31.0	49.4	-	-	-
Intercarotid space (mm)	16.8	16.1	17.6	17.0	4.2	-
Optic chiasm (mm)	13.1	12.7	13.4	13.0	1.8	0.001
Sphenoid sinus width (mm)	28.4	27.8	29.0	29.0	3.2	0.002

The mean age of the participants was 49.1 years (95% CI: 45.6 - 52.5). The Shapiro-Wilk test for normality yielded a p-value of 0.033, indicating a deviation from normal distribution. Regarding gender distribution, 40.2% of the participants were male. The measurements of anatomical structures revealed a mean intercarotid space width of 16.830 mm (95% CI: 16.051 - 17.609) with a standard deviation of 4.161. The Shapiro-Wilk test indicated normal distribution (p = 0.350). The width of the optic chiasm averaged 13.063 mm (95% CI: 12.717 - 13.408). Similar to the male distribution, the Shapiro-Wilk test showed non-normality (p < 0.001). The SS width (SSW) was measured at 28.366 mm on average (95% CI: 27.759 - 28.973).

**Figure 2:**
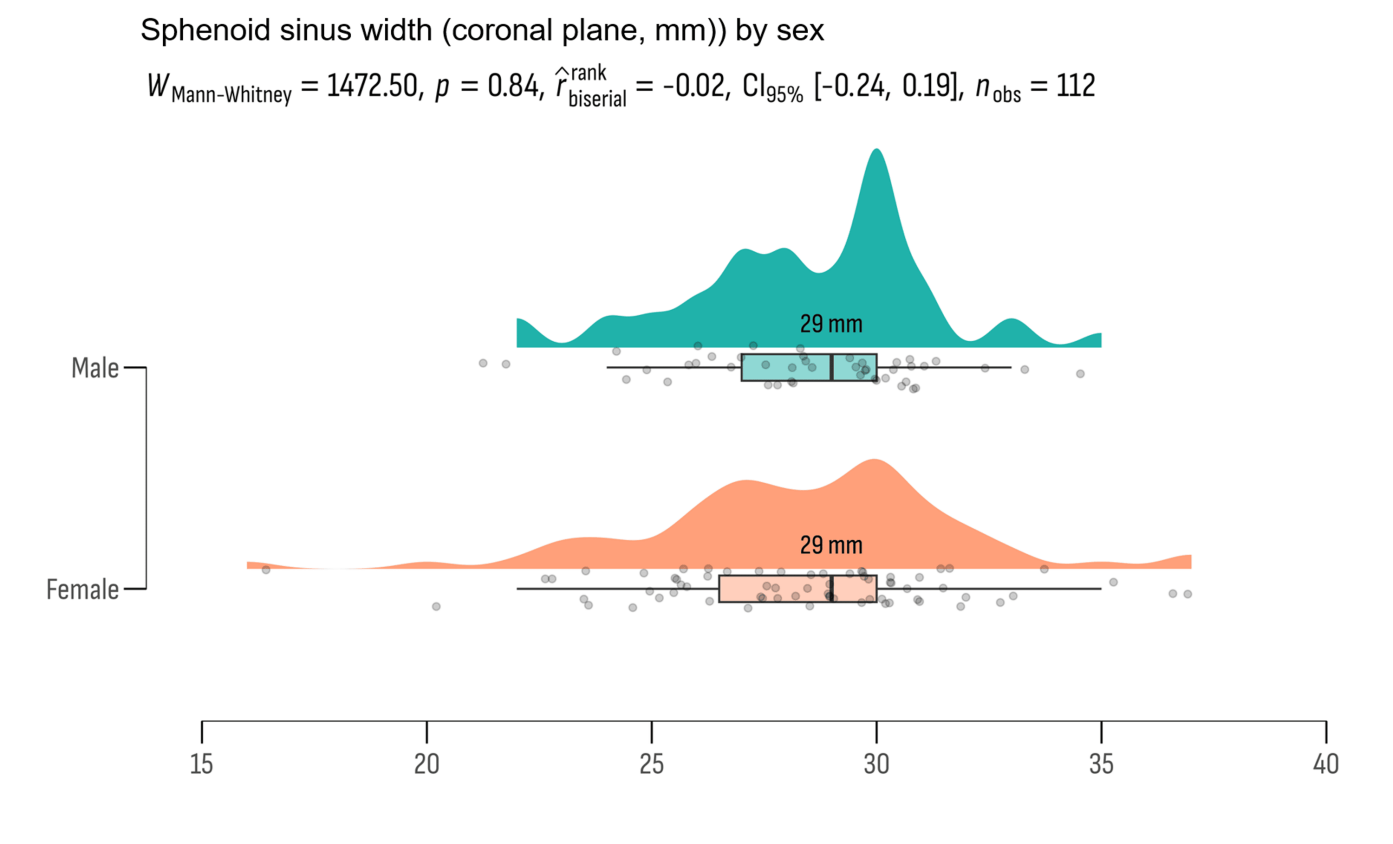
The mean values of sphenoid sinus width represented in millimeters

**Figure 3:**
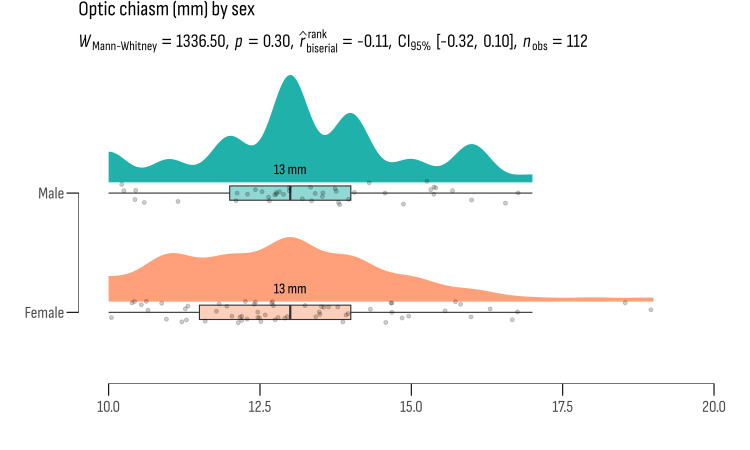
The mean values of optic chiasm width represented in millimeters

**Figure 4:**
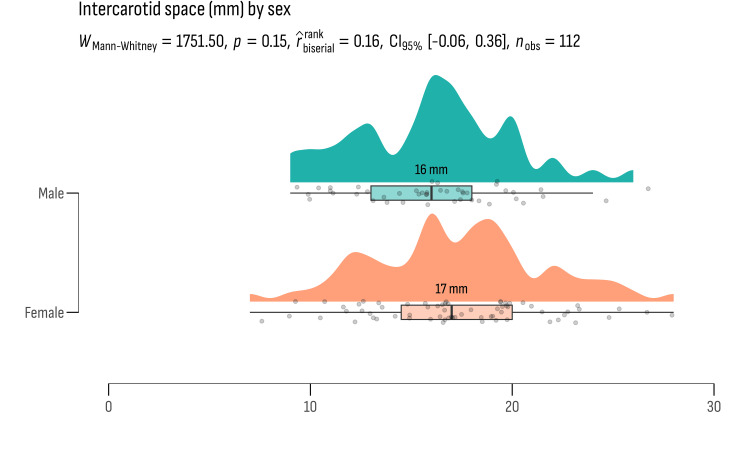
The mean values of intercarotid space represented in millimeters

**Figure 5:**
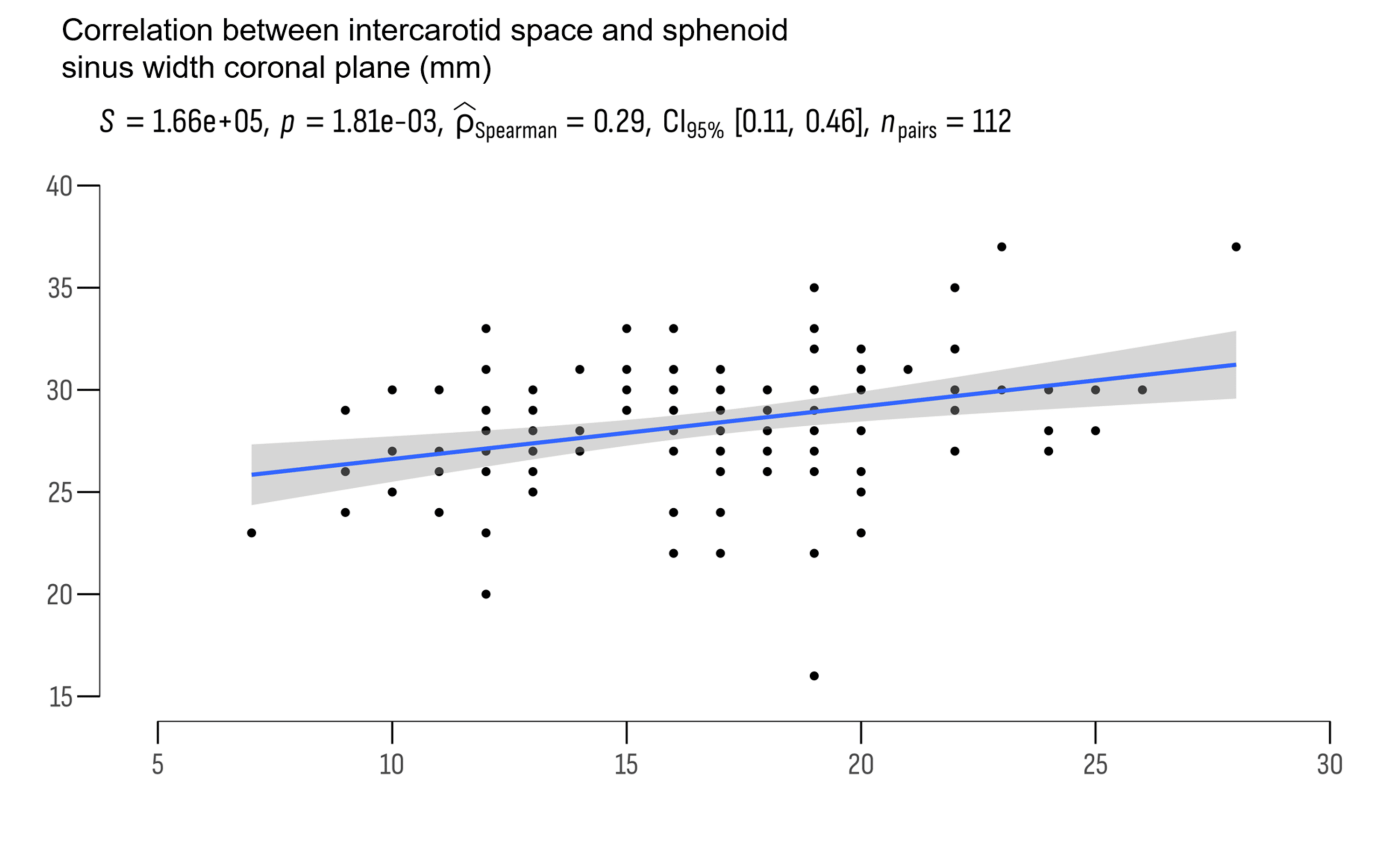
The relationship between the sphenoid sinus width and the intercarotid space

We also conducted independent sample t-tests to explore potential gender differences in key anatomical measurements among the study participants. The results from this analysis are presented in Figures 2-5.

Age distribution between gender groups did not show statistically significant differences (t = -0.911, df = 110, p = 0.364), suggesting similar age profiles across genders in the study cohort. Similarly, anatomical measurements such as the intercarotid space width, optic chiasm width, and SSW did not exhibit significant differences between genders. The intercarotid space width showed a mean difference of 1.508 cm (p = 0.134), the optic chiasm width had a mean difference of -0.749 mm (p = 0.456), and the SSW had a mean difference of -0.268 mm (p = 0.789). These results imply that anatomical variations in these structures do not significantly differ between males and females in the study population.

Effect sizes (Cohen's d) were computed to quantify the practical significance of these differences. The effect sizes ranged from very small (Cohen's d = -0.0516 for SSW) to moderate (Cohen's d = 0.2906 for intercarotid space), indicating minimal to moderate practical relevance of the observed differences.

The correlation matrix (Table 2) explores the relationships among key anatomical measurements and age within the study cohort using Spearman's rho coefficients.

**Table 2:** Correlation matrix of anatomical measurements and age using Spearman’s Rho coefficients Note. *p <.05; **p <.01; ***p <.001

Anatomical structures measured	Intercarotid space (mm)	Optic chiasm (mm)	Sphenoid sinus width (mm)	Age
Intercarotid space (mm)	-	-	-	-
Optic chiasm (mm)	0.105	-	-	-
Sphenoid sinus width (mm)	0.292 **	0.058	-	-
Age	-0.109	0.070	-0.072	-

Intercarotid space width and SSW exhibited a statistically significant positive correlation (Spearman's rho = 0.292, p = 0.002), indicating that individuals with wider SS spaces tended to have larger intercarotid. No significant correlations were found between optic chiasm width and the other variables (intercarotid space, SSW, age), suggesting that optic chiasm dimensions did not correlate with these anatomical features or age within the study population. Age showed a slight negative correlation with intercarotid space width (Spearman's rho = -0.109, p = 0.252) and SSW (Spearman's rho = -0.072, p = 0.453), although these correlations were not statistically significant. This indicates that age may have minimal influence on the dimensions of intercarotid space or SS within this cohort.

## Discussion

The sphenoid bone is located in the middle of the skull base. The body, the corpus ossis sphenoidalis, represents the central part of the bone. Its upper surface is slightly concave and forms the Turkish saddle-the sella turcica. It consists of a median fossa, the fossa hypophysealis, bounded in front by the tuberculum sellae and behind by the dorsum sellae with paired processes, the processus clinoideus anterior et posterior. Lateral to the sella turcica is the sulcus caroticus and lingula sphenoidalis, and anterior to the tuberculum sellae is the sulcus prechiasmaticus, which laterally reaches the canalis opticus. The posterior surface of the body fuses with the pars basilaris of the os occipitale and forms the posterior part of the cranial base. The anterior surface of the body encloses the nasal cavity. In the middle of it is the crista sphenoidalis, which ends in the rostrum sphenoidale. To the side of the crest is a ridge, the concha sphenoidalis, and a paired opening, the apertura sinus sphenoidalis, which leads to the body cavity, the sinus sphenoidalis. The sinus sphenoidalis is divided into two cavities by a septum, septum sinuum sphenoidalium. In infancy, the entrance of the SS is narrowed by a single bony lamella of triangular-shaped sphenoid bone located in the middle of the skull base. The body, the corpus ossis sphenoidalis, represents the central part of the bone. Its upper surface is slightly concave and forms the Turkish saddle-the sella turcica. It consists of a median fossa, the hypophysial fossa, bounded in front by the tuberculum sellae and behind by the dorsum sellae with paired processes, the processus clinoideus anterior et posterior. Lateral to the sella turcica is the sulcus caroticus and lingula sphenoidalis, and anterior to the tuberculum sellae is the sulcus prechiasmaticus, which laterally reaches the canalis opticus. The posterior surface of the body fuses with the pars basilaris of the os occipitale and forms the posterior part of the cranial base. The anterior surface of the body encloses the nasal cavity. In the middle of it is the crista sphenoidalis, which ends in the rostrum sphenoidale. To the side of the crest is a ridge, the concha sphenoidalis, and a paired opening, the apertura sinus sphenoidalis, which leads to the body cavity, the sinus sphenoidalis. The sinus sphenoidalis is divided into two cavities by a septum, septum sinuum sphenoidalium. In infancy, the entrance of the SS is narrowed by a single bony lamella of triangular shape [1-5].

The SS varies in size, shape, and degree of pneumatization (Hamberger et al. 1961.), and the authors divide them into three types: conchal: a small sinus with no connection to the sellae, presellar: the posterior wall of the sinus reaches the sellae but does not cross the anterior surface of the sella turcica; sellar: the posterior wall of the sinus crosses the anterior wall of the fossa hypophysealis, reaches the bottom of the sellae, but does not exceed the posterior wall of the pituitary fossa [2].

Based on this classification, Hardy and associates have developed a standard for access to the sellar region. The most common sellar type Wang and Bidari subdivided into (1) sphenoid body type: pneumatization does not extend beyond the body of the cuneiform bone; (2) lateral type: the sinus extends laterally; (3) clival type: extension of the sinus posterior to the clivus; (4) lesser wing type: pneumatization extends to the lesser wings; (5) anterior type: the cavity expands anterolateral; (6) combined type: when combining the other types [4].

Sphenoidal sinus is quite variable and within a type. The size of the lateral recesses is not the same on the left and right. The left one is usually significantly larger. In addition to the different types of pneumatization, septa, and other bony formations may be seen in the sinus sphenoidalis and also present obstacles to transsphenoidal access to the sellar region. A spheno-ethmoid cell (Onodi cell) is produced by additional septa in the sinus. The Onodi cell was first described by Adolf Onodi in 1903 [4]. It is the most posterior ethmoidal cell that extends to the SS superior-laterally, very close to the n.opticus [5-7].

Various authors over the years have made measurements of the sphenoidal sinus. The team of Kayalioglu et al. found the following measurements in bone, cadaver, and MRI images: length at the superior portion of the sinus: 13.51 +/- 3.25 mm; length at the inferior portion of the sinus: 24.57 +/- 6.65 mm; height at the anterior portion of the sinus: 21.27 +/- 4.25 mm; height at the posterior portion of the sinus: 14.5 +/- 4.07 mm [8].

The team of Dogan et al. found in 50 studied CTs a sellar form in 82% of cases, a presellar form in 44%, a conchal type in 14%, and a post-sellar type in 2%. The study analyzed data from the left and right halves of the sinus, and the results were not compared with each other [9].

The team of Wiebracht et al. examined CT scans of 90 patients and found the following results: 54% post-sellar type, 37% sellar type, and 9% presellar type. The research team also found that in 34% of posterior sinus pneumatization, the intersinus septum was located above the bony covering of the carotid artery [10].

In functional endoscopies, Jaworek-Troc et al. found the following dimensions in 296 CTs performed: 147 women and 149 men: anterior-posterior 2.65 cm (0.5-4.3 cm); transversal 1.98 cm (0.5-4.9 cm); and vertical 2.1 cm (0.7-3.7 cm) [11].

In 60 CT scans of the head performed by Idowu et al. on a Nigerian population of 37 males and 23 females were found to have the following dimensions presented in Table 3 [12].

**Table 3:** Comparative left and right sphenoid sinus sizes in Nigerian patients Source [12]

Dimensions in various planes	Male	Female	Total
Right anterioposterior	24.8 mm	22.1 mm	23.8 mm
Left anterioposrerior	26.3 mm	25.4 mm	26.0 mm
Right transverse	19.1 mm	17.7 mm	18.6 mm
Left transverse	20.3 mm	19.9 mm	20.1 mm

Crac et al. reported that performing an endoscopic sphenoidotomy may be difficult in the hair position of an enlarged septal ramus. Reaching pathological processes in the case of dorsal type of sinus pneumatization is also difficult. In order to avoid damage to the anatomical elements in the area under consideration, the authors recommend the use of an angled endoscope and a microsurgical transsphenoidal indumentarium in these cases [13].

The SS is located in the body of the cuneiform bone. It is usually divided into two cavities [14, 15]. It is located below the sella turcica and sulcus chiasmaticus. It can also be divided into different numbers of small cavities, which are discontinuous. Through the aperture sinus sphenoidalis, the sinus cavity communicates with the recessus sphenoethmoidalis of the superior nasal passage. At birth, the SS is not pneumatized. Pneumatization begins at two to three years of age. Its development proceeds from three to five to six and 10 years of age with little variability. The sinus develops slowly, forms by the sixth year as a small cavity, and completes its development at the end of the growth period of the body [14].

The SS is lined with a mucous membrane, which is an immediate extension of the mucous membrane of the nasal cavity. It has the same morphology, with some reduction in the amount of cilia, and the lamina propria is considerably thinner. The body of the sphenoidal bone originates from two paired ossification centers: pre-sphenoidal and post-sphenoidal [2-4]. Pneumatization of the sinus progresses anterior to posterior, forming its main cavity. More peripheral aeration in the directions described above results in the corresponding types. The degree of pneumatization and the direction in which it develops plays an important role in determining access to the sellar region. The structure of the sinus also determines the access corridors to the surrounding anatomical elements. The lesser wing type provides the best opportunity for a convenient surgical corridor to the lateral suprasellar region [14, 16]. The lateral recesses can accomplish a more straightforward route to this area via transmaxillary and transsphenoidal access. The clival type of sinus is well suited for a transnasal approach to the posterior cranial fossa because of the thin clivus [3, 14].

In 1960, Hardy introduced the operating microscope. Many opportunities for error arise due to the fact that procedures are performed on a very small anatomic space with unique physiology and structural variabilities. The sinus sphenoidalis increases with age and has a highly variable cavity structure. The latter is located near some important structures: optic nerve, cavernous sinus, internal carotid artery, frontal lobe, ventral surface of the brainstem, cranial nerves III to VI, pituitary gland, and median canal. The course of the canalis pterygoideus along the floor of the SS is also a prerequisite for the damage of the nerve of the same name when its canal opens into the sinus cavity [3, 4, 6, 7, 17].

The internal carotid artery lies in a groove located along the intracranial surface of the body of the cuneiform bone. When a prominent cuneiform recessus is unexpectedly detected, its injury is possible. Although the internal carotid artery is located approximately 3 mm to 7 mm lateral to the sinus wall, it is sometimes possible for the pituitary gland to grow into the cavernous sinus. In 4%-8% of cases, the artery may project into the sinus cavity and create the preconditions for its damage during surgery. The upper wall of the SS is formed by the planum sphenoidale anteriorly and the sella turcica posteriorly. The different positions of the chiasma opticum in the suprasellar region are: in front of the tuberculum sellae, over the diaphragma sellae, and over the dorsum sellae. This positioning of the chiasma can cause damage to the elements in this area [6].

Primary malignant tumors of the SS range from 1% to 2% of sinus tumors, according to literature data. The sinus can also be a site of secondary metastasis from tumors of the mammary gland, lung, kidney, thyroid, or prostate [6, 16].

The sphenoidal sinus has a highly variable septal and cavitary architecture. Accurate preoperative diagnosis is necessary to avoid possible complications, including hemorrhages and other serious problems. The degree and type of SS pneumatization play an important role in providing different surgical accesses to certain structures. Preoperative head axial CTs and MRIs of the pituitary region are of great importance in determining the unique anatomy of each individual patient and delineating the appropriate surgical access route [14, 15].

Surgical approaches to this delicate area involve careful preoperative imaging analysis and good planning of the surgical approach relative to the pathological processes. For this reason, many research studies have been dedicated to studying the microanatomy and variations in the SS and its adjacent neural and vascular structures [18].

## Conclusions

The SS represents an important corridor in reaching pathologic processes located in the sellar and parasellar regions. Knowledge of the anatomical structure and variations in the latter are crucial in planning surgical interventions. The MRI of the brain provides detailed information on the types of SS, the width of the latter, and its relation to the carotid artery, chiasm, and pituitary gland. In the present study, we pooled and compared the results with the world literature in 112 patients who underwent an MRI of the brain and demonstrated a correlation between the width of the SS and the intercarotid space. In this way, we can make a good preoperative plan for what should be the volume of trepanation of the SS without causing iatrogenic trauma to the intact nerve structures. In this way, we can make a good preoperative plan for what should be the volume of trepanation of the SS without causing iatrogenic trauma to the intact nerve structures.
